# Time-Resolved Study of Light-Induced Ground-State Proton Transfer from an Acidic Medium to 4‑Nitrophenolate

**DOI:** 10.1021/acsphyschemau.5c00022

**Published:** 2025-07-28

**Authors:** Leandro Scorsin, René A. Nome, Ricardo F. Affeldt, Fabiano S. Rodembusch, Faruk Nome

**Affiliations:** † Instituto de Química, 201359 Universidade Federal do Rio Grande do Sul−UFRGS. 91501-970 Porto Alegre, Rio Grande do Sul, Brasil; ‡ Instituto de Química, 28132 Universidade Estadual de Campinas−UNICAMP. 13083-970 Campinas, São Paulo, Brasil; § Departamento de Química, 201363 Universidade Federal de Santa Catarina−UFSC. 88040-900 Florianópolis, Santa Catarina, Brasil

**Keywords:** ground-state proton transfer, laser flash photolysis, 4-nitrophenol, protonation kinetics, transient absorption spectroscopy

## Abstract

This work investigates the transient laser-induced formation of 4-nitrophenolate in the ground electronic state and its subsequent proton transfer with acetic acid and water. Laser flash photolysis in the UV–vis region revealed the presence of a deprotonated transient species even at weakly acidic pH. We measured the photoinitiated ground state protonation and deprotonation rate constants of 4-NPO^–^/4-NPOH as a function of acetic acid, pH, and temperature. This study demonstrates a simple approach to analyzing fast competing bimolecular proton transfer reactions under nonequilibrium conditions in the ground state.

## Graphical abstract

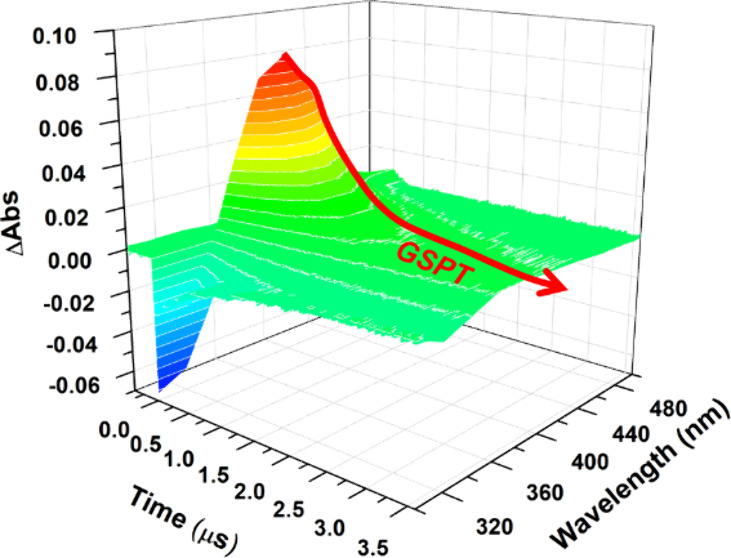

Many chemical reactions in both natural and synthetic systems occur via thermally activated pathways in the electronic ground state. Investigating the dynamics of such processes, especially under nonequilibrium conditions, remains challenging and less explored compared to excited-state reactions and often requires time-resolved techniques capable of resolving reaction steps on the nanosecond to microsecond time scale.^1^ In this context, laser flash photolysis^2,3^ provide a powerful approach to probe these ground-state reactions with high temporal resolution,^4,5^ enabling the direct observation of transient intermediates and subsequent bimolecular events such as protonation and deprotonation.

Among model systems, phenolic photoacids like 4-nitrophenol (4-NPOH) stand out for their well-characterized reactivity and favorable spectral properties.^6–12^ Moreover, 4-NPOH has been applied in mechanistic investigations of photoacidity, p*K*_a_ shifts, and solvation effects.^13–20^ This combination of well-defined reactivity and favorable spectral characteristics establishes 4-NPOH as a reliable, robust and versatile platform for probing ground-state proton transfer (GSPT) processes. Upon light excitation, 4-NPOH undergoes a cascade of nonradiative transitions including internal conversion and intersystem crossing (ISC), ultimately leading to the efficient formation of the ground-state phenoxide anion (4-NPO^–^), which can be easily monitored via its strong UV–vis absorption, enabling the study of proton transfer kinetics on time scales ranging from nanoseconds to milliseconds.^21–24^ A comprehensive investigation by Gosh et al.^25^ characterized the complete photophysical behavior of 4-NPOH, from femtoseconds to microseconds, and conclusively identified the long-lived transient as the ground-state phenoxide anion. Importantly, their work ruled out direct deprotonation from the singlet excited state, showing instead that ISC to the triplet manifold procedes proton transfer and ground-state anion generation. Building on these insights, we do not aim to propose a new mechanistic model for 4-NPOH, but rather to exploit its well-established photoacid cycle as a framework to probe ground-state proton transfer (GSPT) reactions under nonequilibrium conditions. Specifically, we investigate the kinetics and activation parameters of bimolecular protonation/deprotonation processes involving acetic acid and acetate in buffered aqueous solutions. By using laser flash photolysis to monitor these reactions in real time, we demonstrate the broader applicability of this technique to track thermally activated chemical events initiated by photoexcitation. The photophysical cascade begins with excitation of 4-NPOH to its singlet excited state (4-NPOH→4-NPOH*, [S_1_-ππ*]),^26^ followed by internal conversion to S_1_[ππ*]→S_1_[nπ*], and finally causing an ISC S_1_[nπ*]→T_2_[ππ*].^27^ Before deprotonation an internal conversion in 4-NPOH* is observed (T_2_[ππ*]→T_1_[ππ*]). Proton transfer can then proceed either directly from T_1_[ππ*] to the ground-state S_0_ (4-NPOH*→4-NPO^–^) or via an excited state proton transfer in the triplet state T_1_[ππ*] (4-NPOH*→4-NPO^–^*).^25^ For 4-NPO^–^* at T_1_[ππ*], a new ISC and other nonradiative relaxations (such as vibrational energy transfer) lead to the formation of 4-NPO^–^ in S_0_.^28^ Efficient generation of this anion enables further ground-state chemistry to be studied, including its reaction with water, acetic acid, or other species under nonequilibrium conditions, as illustrated in Scheme 1. Finally, 4-NPOH’s nonfluorescent nature and rapid vibrational relaxation favor efficient conversion to 4-NPO^–^. When external reactants are present, new GSPT pathways emerge, allowing for systematic exploration of acid–base dynamics and the influence of weak acids and their conjugate bases. Altogether, this work reinforces the utility of 4-NPOH as a robust model for exploring photoinduced formation of transient ground-state species and their role in fundamental proton transfer processes.^29–31^

**Scheme 1:**
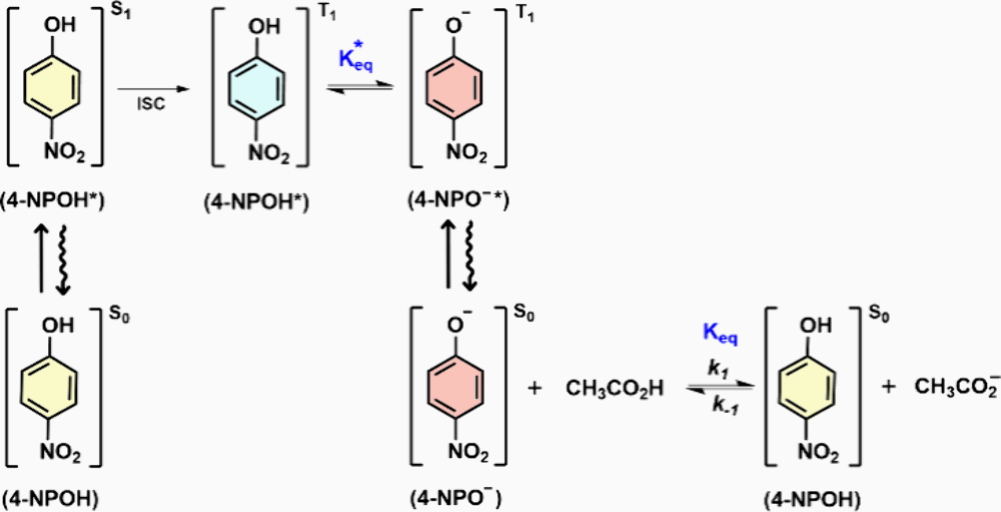
Photochemical Pathway for the Formation of Ground-State 4-Nitrophenolate (4-NPO^–^) via Intersystem Crossing (ISC) and Proton Transfer from 4-Nitrophenol (4-NPOH), Followed by Bimolecular Ground-State Protonation

Using the laser flash photolysis technique, a short laser pulse is applied to an acidic solution containing 4-NPOH, initiating the photocycle. Pulsed white light is then used to probe the resulting kinetics, leading to the formation of the transient species 4-nitrophenolate, which, in this study, may react with water or acetic acid to return to its neutral form. Previous studies on photoacids have characterized transient species as either radicals^32^ or cation radicals.^33^ Although a similar technique was employed, the medium in which the photoacid was studied differs from that used in our work. While those studies primarily focused on excited state hydrogen transfer (ESHT),^32^ the present investigation centers on ground state proton transfer (GSPT).

Figure 1 presents the time-resolved transient absorption spectra following 4-NPOH excitation at 266 nm, recorded between 300 and 500 nm. The key experimental features are illustrated in Figure 1 (bottom): a fast decay (9 ns) of the ground-state bleaching signal near 300 nm, corresponding to the immediate disappearance of the neutral 4-NPOH species, and the fast and efficient formation of 4-NPO^–^ in the ground state at 400 nm (29 ns). Subsequently, equilibrium is established, leading to the protonation of 4-NPO^–^, as indicated by a decrease in absorbance at 400 nm, concurrent with an increase in absorbance at 300 nm, which is consistent with the regeneration of 4-NPOH.

**Figure 1:**
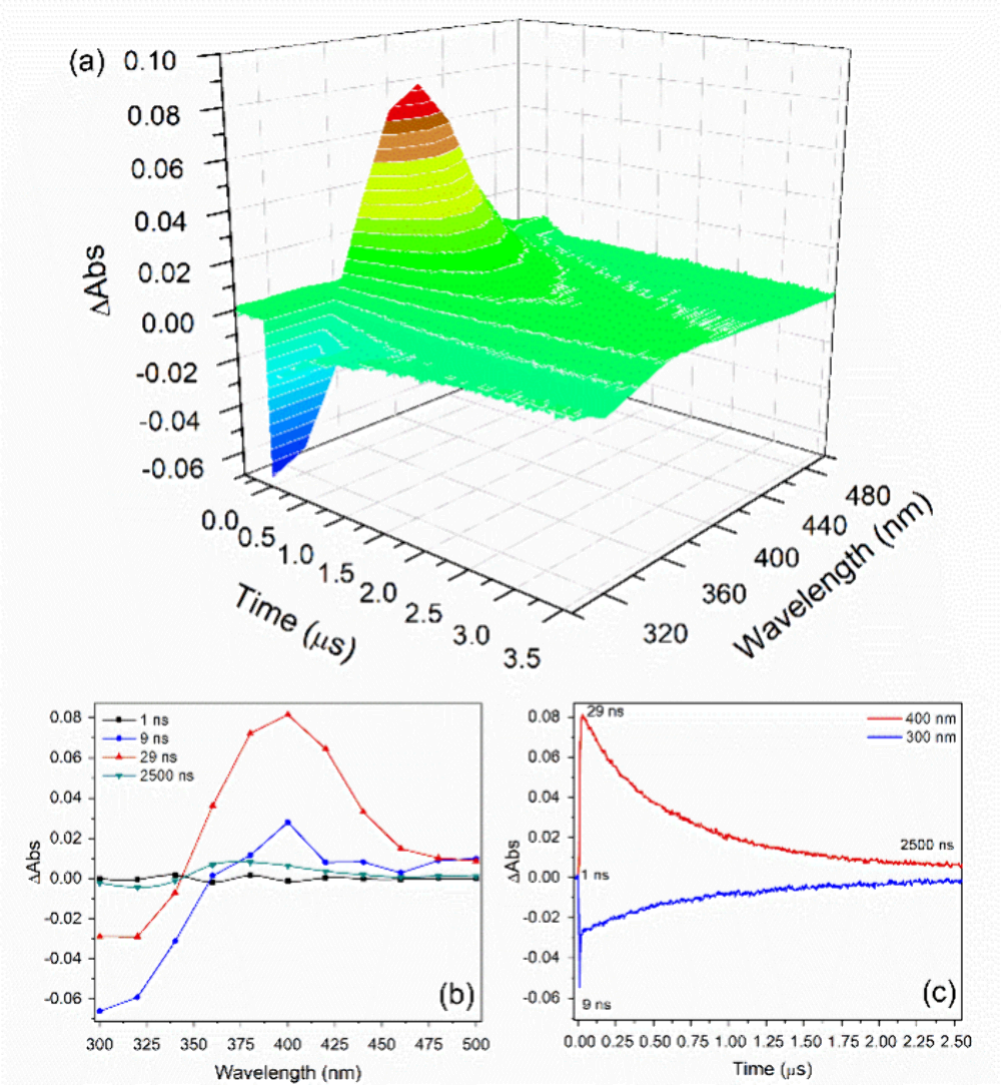
(a) Time-resolved transient absorption spectra following 4-NPOH (c_0_ = 2.0 × 10^–5^ mol·L^–1^) excitation at 25 °C and pH 4.6. (b) Transient absorption spectra at 1 ns (black), 9 ns (blue), 29 ns (red), and 2500 ns (green). (c) Transient absorption signals as a function of time at probe wavelengths of 300 nm (blue) and 400 nm (red).

Figure S1 in the Supporting Information (SI) presents the pH-dependent kinetics of 4-NPO^–^ protonation with the pH range of 4.0–5.2, demonstrating faster decay under more acidic conditions. Each plot corresponds to a specific acetic acid concentration, ranging from 0.5 × 10^–3^ to 1.0 × 10^–2^ mol·L^–1^, showing that at a given pH, the decay rate increases with rising acetic acid concentration. Nonlinear least-squares fitting of the data in Figure S1 provides the observed rate constants plotted in Figure 2. The highest observed rate constants were obtained at the lowest pH (4.0) and the highest AcOH concentration (1.0 × 10^–2^ mol·L^–1^), with a linear increase in the rate constant across all examined pH values. The intercepts of the lines in Figure 2, corresponding to [AcOH] = 0, were used to characterize the rate constants as a function of H_3_O^+^ concentration.

**Figure 2:**
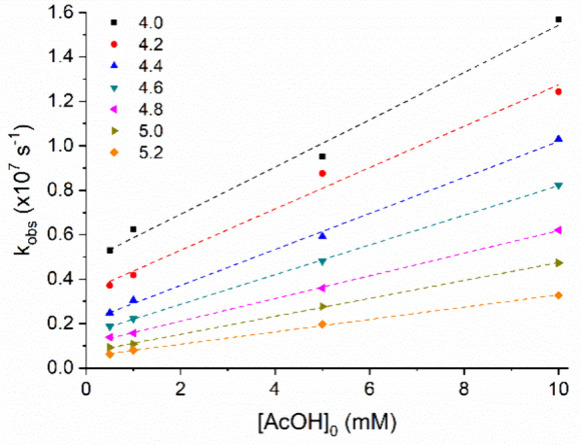
Observed rate constant (*k*_obs_) as a function of increasing AcOH concentration and pH variation for 4-NPO^–^.

The results align with Eigen’s^34^ theoretical treatment, which describes the protonation of 4-nitrophenolate by water molecules as a fast, direct second-order reaction with a rate constant of approximately 3.5 × 10 ^10^ L·mol^–1^·s^–1^).^35^ In contrast, when the medium is slightly acidic due to the addition of a weak acid such as acetic acid, the reaction also proceeds via a protolysis mechanism. In this case, proton transfer to nitrophenolate can occur via either carboxylic acid molecules or hydronium ions. Equation accounts for the pH-dependent molar fraction of acetic acid and acetate species present in solution:

$$k_{\mathrm{obs}}=\left(k_{\mathrm{AcOH}}^{P}\chi_{\mathrm{AcOH}}+k_{\mathrm{AcO}}^{D}\chi_{\mathrm{AcO}}\right){\left[\mathrm{AcOH}\right]}_{0}+k_{H}^{P}\left[H_{3}O^{+}\right]+k_{H}^{D\prime }$$

The linear relationship observed is consistent with eq 1, where the intercept represents the deprotonation constant (k_H_^D^’ = k_H_^D^[H_2_O], [H_2_O] = 55.5 mol·L^–1^) of 4-NPOH, while the slope corresponds to the rate constant for the protonation (k_H_^P^) of 4-NPO^–^ by hydronium ions, as shown in Figure 3, with values provided in Table 1.

**Figure 3:**
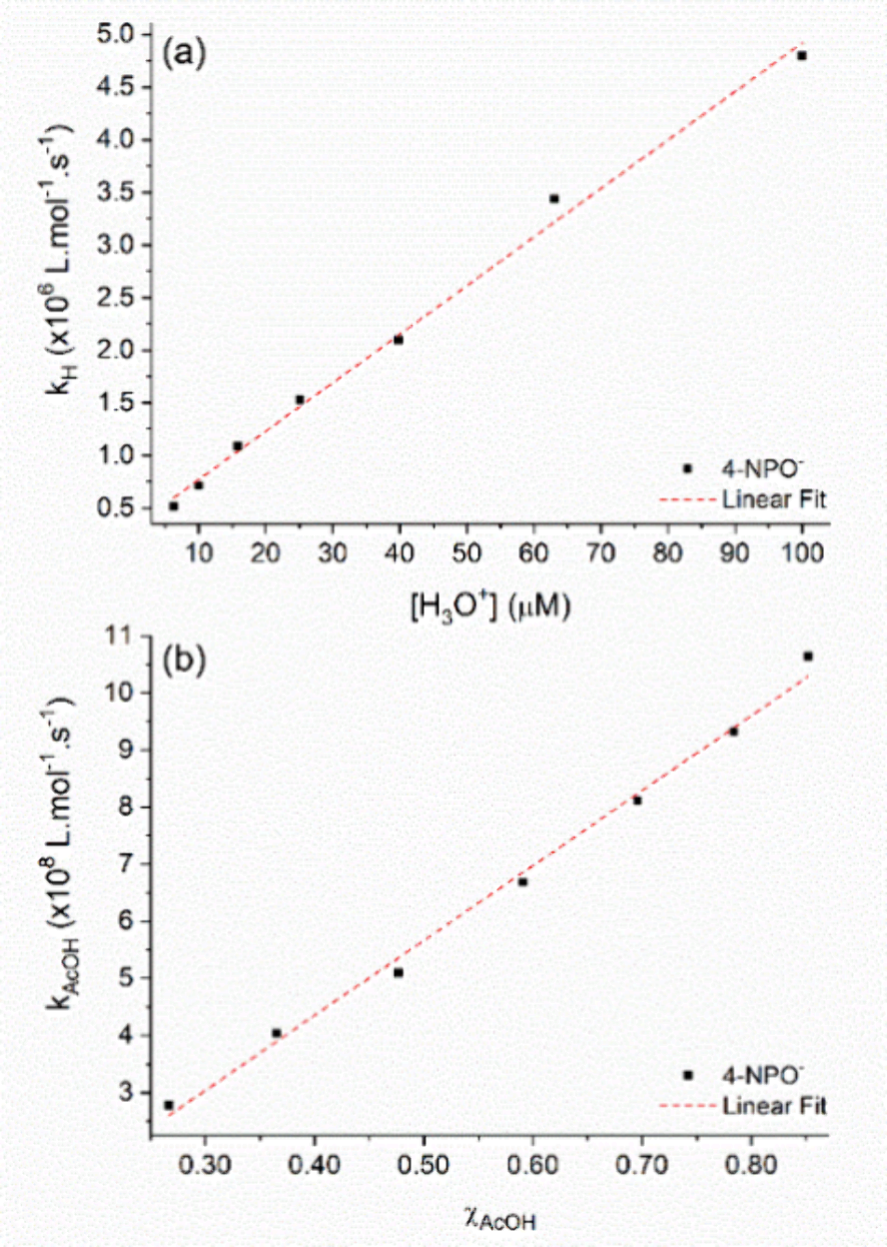
(a) Rate constant (k_H_) for the reaction of 4-NPO^–^ with hydronium ions as a function of [H_3_O^+^] and (b) rate constant (k_AcOH_) as a function of the increasing molar fraction of AcOH.

**Table 1:** Constant Protonation (P) and Deprotonation (D) Rates for the Reactions of the 4-NPO^–^/4-NPOH pK_a_ = 7.15) and Acetic Acid Buffer Media

k_AcOH_^P^ (L**·**mol^–1^ **·**s^–1^)	k_AcO_^D^ (L**·**mol^–1^ **·**s^–1^)	k_H_^P^ (L**·**mol^–1^ **·**s^–1^)	k_H_^D^′ (s^–1^)
(1.31 ± 0.05) × 10^9^	(9.0 ± 0.3) × 10^7^	(4.61 ± 0.15) × 10^10^	(3.1 ± 0.8) × 10^5^

Figure 3 presents the second-order rate constants derived from the slope of the plots in Figure 2 as a function of the molar fraction of AcOH. The observed linear relationship enables the calculation of the deprotonation rate constant (k_AcO_^D^) and the protonation constant (k_AcOH_^P^) for the reaction with acetic acid, along with the corresponding rate constant values. Another important factor influencing the observed kinetics is the ionic strength of the solution. Since our experiments were conducted under low ionic strength conditions, we acknowledge that the rate constant for protonation may be subject to significant variation due to electrostatic interactions between charged species. This is particularly relevant for the 4-NPO^–^ anion reacting with H_3_O^+^ or AcOH. In fact, the slight curvature observed in Figures 3a and 3b may reflect such ionic strength effects, as well as specific buffer interactions or deviations from ideal kinetic behavior.

These calculated values accurately reproduce the observed rate constant (black dots in Figure 4) as a function of pH and acetic acid concentration. The continuous red line represents the predicted values, obtained using the rate constants listed in Table 1. The agreement between experimental and calculated values confirms the validity of Eigen’s treatment and demonstrates the reliability of the experimental data obtained through this new approach. Notably, this study was conducted in a slightly acidic medium (pH 4.0–5.2), where the reaction proceeds concurrently with protolysis.^34,35^ Our measured second-order rate constants are consistent with previously reported values for the nitrophenolate-to-nitrophenol reaction, which range from 3.5 × 10^10^ to 5.2 × 10^10^ L·mol^–1^·s^–1^ between pH 3 and 5.^25^

**Figure 4:**
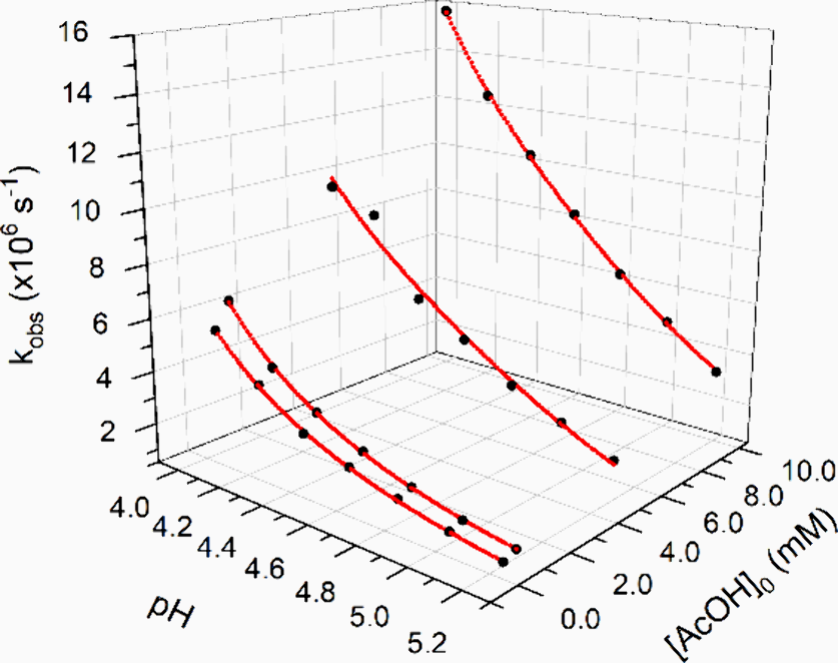
Nonlinear curve fits (red lines) of *k*_obs_ as a function of pH and [AcOH] for 4-NPO^–^. The black spheres represent the observed *k*_obs_values.

These calculated values accurately reproduce the observed rate constant (black dots in Figure 4) as a function of pH and acetic acid concentration. The continuous red line represents the predicted values, obtained using the rate constants listed in Table 1. The agreement between experimental and calculated values confirms the validity of Eigen’s treatment and demonstrates the reliability of the experimental data obtained through this new approach. Notably, this study was conducted in a slightly acidic medium (pH 4.0–5.2), where the reaction proceeds concurrently with protolysis.^34,35^ Our measured second-order rate constants are consistent with previously reported values for the nitrophenolate-to-nitrophenol reaction, which range from 3.5 × 10^10^ to 5.2 × 10^10^ L·mol^–1^·s^–1^ between pH 3 and 5.^25^

The rate constants for protonation by H_3_O^+^ are higher than those for AcOH. Consequently, the acid dissociation constant can be determined as K_a_ = k^D^/k^P^, using the values from the last two columns in Table 1 and the relation k_H_^D^′ = k_H_^D^[H_2_O], k_H_^D^ = 5.6 x10^3^ L·mol^–1^·s^–1^. This yields a p*K*_a_ value of 6.92. Ideally, rate constants obtained from relaxation to equilibrium should reproduce the known p*K*_a_ of 4-NPOH (7.15).^36^ The slight discrepancy between the calculated and literature values is attributed to the previously discussed competition between protonation mechanisms (protolysis) and direct proton transfer in this pH range, where both AcOH and H_3_O^+^ are involved. In our work, changes in pH and AcOH concentration caused a slight variation in the ionic strength of the medium. This variation becomes insignificant for obtaining the protonation and deprotonation constants, since, in the literature, greater implications of ionic strength maintain similarity between the decay times for 4-NPOH in the ground-state (e.g., absence and presence of 1 M of salt, at pH 5).^25^ Previous studies have shown that salts of weak acids (e.g., fluoride and acetate) with p*K*_a_ values between ground and excited state acidity constants can react with the photoacid.^37^ As expected, the rate constant for proton transfer between AcOH and 4-NPO^–^ increases with temperature, exhibiting the expected dependence according to both the Arrhenius (Figure S2 in the Supporting Information) and Eyring (Figure S3 in the Supporting Information) models. The calculated activation parameters are presented in Table 2.

**Table 2:** Arrhenius and Activation Parameters of 4-NPO^–^ ^a^

*E*_a_	log *A*	ΔH^⧧^	ΔG^⧧^	ΔS^⧧^
2.61	8.49	2.01	8.48	–21.70

a *E*_a_, ΔH^⧧^, and ΔG^⧧^ are presented in kcal·mol^–1^ and ΔS^⧧^ is presented in cal·mol^–1^·K^–1^.

The activation energy (*E*_a_) is relatively low (2.61 kcal·mol^–1^), and the pre-exponential factor (log A = 8.49) is also modest, consistent with the fast nature of proton transfer reactions involving anionic species such as 4-nitrophenolate and weak acid donors like acetic acid. The positive activation enthalpy (ΔH^⧧^ = 2.01 kcal·mol^–1^) reflects the intrinsic energy required to reorganize solvent and solute species and overcome the transition state barrier during the protonation event. The large and negative activation entropy (ΔS^⧧^ = −21.7 cal·mol^–1^·K^–1^) indicates a significant decrease in disorder upon forming the transition state. This entropic penalty is typical of bimolecular reactions, particularly those requiring specific spatial alignment and hydrogen bonding interactions between donor and acceptor. In such cases, both species must diffuse and orient precisely in a short time window, imposing strict geometrical constraints. The resulting decrease in degrees of freedom contributes to the reduction in entropy. Although the observation that the Gibbs free energy of activation (ΔG^⧧^ = 8.48 kcal·mol^–1^) exceeds the activation enthalpy is a mathematical consequence of the negative entropy, this finding also reinforces the mechanistic interpretation: the reaction rate is significantly influenced not only by the energetic barrier but also by entropic contributions, especially diffusion, solvent reorganization, and molecular orientation in the encounter complex. Altogether, the thermodynamic parameters support a bimolecular mechanism governed by both energetic and entropic constraints under mildly acidic aqueous conditions.

It is important to clarify that the Arrhenius and activation parameters were obtained from the temperature dependence of the observed first-order rate constant at pH 4.2. A simple constant describes a system involving multiple equilibria and reversible protonation steps, these parameters should be interpreted as apparent activation parameters that reflect the overall observed process, rather than elementary steps. While they may not isolate individual reaction barriers, the values provide useful insights into the global energy landscape of the protonation process under acidic conditions, including the enthalpic and entropic contributions to the observed kinetics.

These parameters indicate a small activation barrier, which is consistent with expectations and previous studies.^22^ All the experimental evidence presented here, including rate constants and energy values, supports an Eigen-type proton transfer via a protolysis mechanism involving the 4-nitrophenolate anion.^34,35^ While viscosity is known to influence proton transfer kinetics, particularly through its effect on diffusion-controlled steps, this parameter was not systematically investigated in the present study. The preliminary observations on viscosity effects remain inconclusive under the current experimental conditions.

In conclusion, the methodology presented here—combining time-resolved transient absorption spectroscopy (TAS) with controlled acid–base conditions—offers a robust platform for investigating photoinduced ground-state proton transfer in solution. Using 4-NPOH as a model photoacid system, we measured time-resolved transient absorption spectra following its excitation, systematically varying pH, AcOH concentration, and temperature. These time-resolved spectroscopy data were used to investigate the protonation and deprotonation reactions of 4-nitrophenolate and acetic acid. Overall, our experimental approach enables us to characterize the ground-state reaction between 4-nitrophenolate and acetic acid, even at pH values significantly lower than the p*K*_a_ of 4-NPOH. Beyond 4-nitrophenol, this approach can be extended to other phenolic systems and hydroxyaromatics, including nitrophenol derivatives, substituted catechols, and salicylic acid analogs. Moreover, TAS holds potential for probing intramolecular proton transfer events and solvent-mediated mechanisms, particularly in systems where the time scale of proton exchange is accessible within the nanosecond to microsecond range. These extensions would allow for a more comprehensive mechanistic understanding of photoacid behavior and proton-coupled dynamics in both synthetic and biological contexts. Therefore, our findings contribute not only to the characterization of a model system but also to the development of generalized tools for studying fast ground-state reactivity under light-induced nonequilibrium conditions.

## Supplementary Material



## Notes

Details about experimental section, supporting data, and effect of temperature (PDF)
