# Supplementary material for: Time-Resolved Study of Light-Induced Ground-State Proton Transfer from an Acidic Medium to 4‑Nitrophenolate
Source: ACS Phys Chem Au. 2025 Jul 28;5(5):450–5. doi: 10.1021/acsphyschemau.5c00022 (PMC12464748; doi:10.1021/acsphyschemau.5c00022)
Supplement: Supplementary file 1 [file pg5c00022_si_001.pdf]

Supporting Information

Time-Resolved Study of Light-Induced Ground-State Proton Transfer from an Acidic Medium to 4-Nitrophenolate

Leandro Scorsin,[a,*] René A. Nome,[b] Ricardo F. Affeldt,[c] Fabiano S. Rodembusch,[a,*] and Faruk Nome[c,≠]

[a]Institute of Chemistry, Federal University of Rio Grande do Sul, Porto Alegre, RS 91501-970, Brazil.

[b]Institute of Chemistry, State University of Campinas (UNICAMP), Campinas, SP 13083-970, Brazil.

[c]Department of Chemistry, National Institute of Catalysis, Federal University of Santa Catarina, Florianópolis, SC 88040-900, Brazil.

[≠] Deceased - 24th September 2018

Experimental Section

All chemicals were purchased from Sigma-Aldrich. 4-nitrophenol was dissolved in water ([4-NPOH] = 1.0 x 10^{-5} mol·L^{-1}). Acetic acid was used in sodium acetate hydrate form ($CH_3COONa·3H_2O$) with addition of strong acid solution until values of pH above and below its pK_a (4.76). The measurements were performed using a pH-meter equipped with a Hanna HI1330 reference pH electrode (Hanna Instruments). The medium pH was varied from 4.0 to 5.2 and acetic acid concentration ranged from 0.5x10^{-3} to 1.0x10^{-2} mol·L^{-1}. In order to obtain the activation parameters, a temperature variation of 15 to 45 ^{o}C was performed at 1.0 mM acetic acid concentration and pH 4.2. Acetic acid was chosen because it does not present absorption in the UV-vis excitation range of the equipment. Water was deionized, reaching resistance >17 MΩ. All experiments were conducted with strict temperature control using thermostated systems both in the preparation of the buffers and in the analysis.

Laser Flash Photolysis Measurements

In this work, we used an Applied Photophysics, LKS80 Laser Flash Photolysis model for nanosecond transient absorption acquisitions on timescales up to

microseconds with 3 ns time resolution. The pump source is the fourth harmonic of a Nd:YAG laser giving about 50 mJ at a wavelength of 266 nm. We estimate that self-focusing, self-diffraction, and multi-photon absorption contribute negligibly to the observed signals. The sample compartment was positioned for focusing the excitation laser pump at 90 degrees geometry with respect to the probe. The probe light source was produced by Q-switching a Xenon Lamp with 150 W per pulse. Transient absorption signals were detected by a five-stage photomultiplier R928 operating from 190 nm to 920 nm and rise time of 3 ns, placed after the monochromator in Czerny-Turner optical configuration with slits of 0.1 to 8.0 mm spacing. All measurements were performed in a fluorescence cuvette with an optical path length of 1.0 cm. Measurement results were observed on the oscilloscope, Tektronix model DPO7000, and analyzed with the LKS Pro-date software.

Data Supporting

Figure S1 shows pH-dependent kinetics of $4\text{-}NPO^-$ protonation in the pH range from 4.0 to 5.2, with faster decay under more acidic conditions. Each plot corresponds to a single acetic acid concentration, ranging from 0.5×10^{-3} to 1.0×10^{-2} $mol{\cdot}L^{-1}$ such that, for each pH, faster decays are observed with increasing acetic acid concentration. The observed rate constant (k_{obs}) is obtained from the decay of the curves (pseudo-first-order).

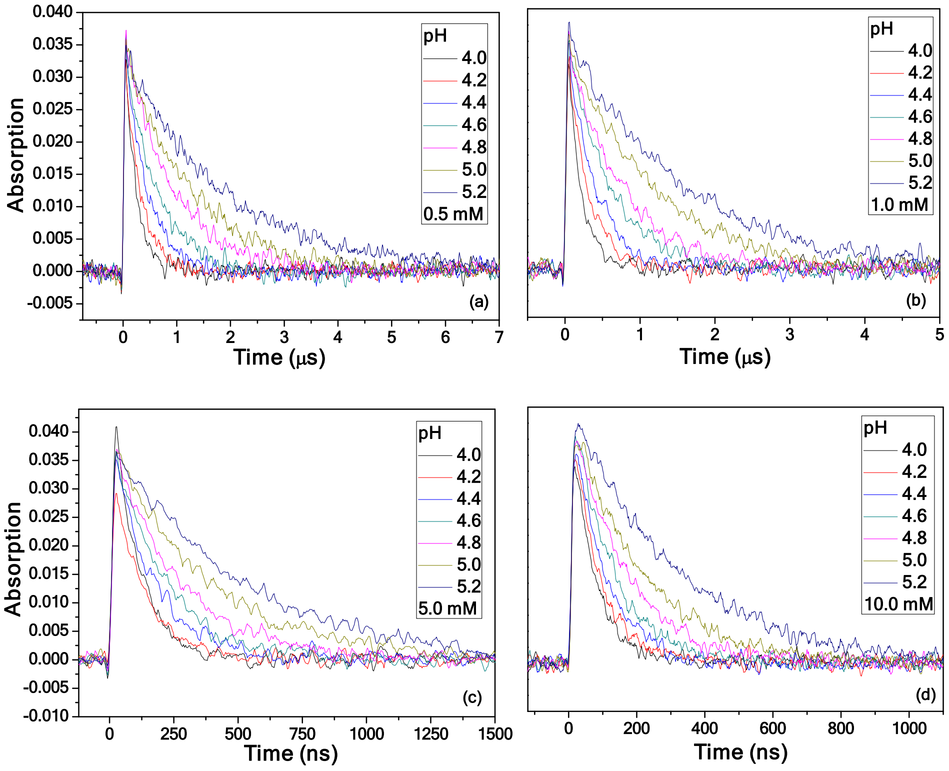

Figure S1. Decay of 4-NPO$^-$ in [AcOH] (a) 0.5x10^{-3}, (b) 1.0x10^{-3}, (c) 5.0x10^{-3}, and (d) 1.0x10^{-2} mol·L^{-1}, pH 4.0-5.2 at 400 nm.

Derivation of the equation for k_{obs}

To derive Equation 1 it is necessary to initially consider that the observed rate constant could be expressed as a function of a proton donor and proton acceptor species, ([PD] and [PA], respectively) and governed by the protonation (slope) and deprotonation (intercept) constants (k^p and k^d, respectively).

$$k_{obs} = k^p[PD] + k^d[PA]$$

As a function of pH, we can adopt H_3O^+ as PD and H_2O as PA. Direct substitution in the equation would justify the change to the constants as a function of H^+ (k_H^P). The constant k_H^D can be interpreted by an apparent constant $k_H^{D}{'}=k_H^D[H_2O]$.

$$k_{obs} = k_H^P[H_3O^+] + k_H^{D}{'}$$

However, this system is also influenced by acetic acid as source of H^+ ions and needs to be added to the equation. The concentrations of the AcOH and AcO$^-$

species, at equilibrium, will be the respective protonation and deprotonation constants (k_{AcOH}).

$$k_{obs} = k^{P}_{AcOH}[AcOH] + k^{D}_{AcO}[AcO^{-}] + k^{P}_{H}[H_3O^{+}] + k^{D}_{H}{}'$$

Finally, the initial concentration of acetic acid $[AcOH]_0$ will be distributed in equilibrium between acid and base. For this to be done correctly, we will be implemented the molar fraction (*X*) as a function of the pH of the medium and the pK_a of AcOH (4.76).

$$\chi_{AcOH} + \chi_{AcO} = 1$$

$$\frac{1}{1 + 10^{(pH - pK_a)}} + \frac{1}{1 + 10^{(pK_a - pH)}} = 1$$

$$k_{obs} = (k^{P}_{AcOH}\chi_{AcOH} + k^{D}_{AcO}\chi_{AcO})[AcOH]_0 + k^{P}_{H}[H_3O^{+}] + k^{D}_{H}{}'$$

Effect of Temperature

Figure S2 shows the linear fit for the Arrhenius parameters for 4-NPO^{-} and shows the value of activation energy E_a and the pre-exponential parameter *A*.

$$k_{obs} = Ae^{-(E_a/RT)}$$

EE_a **(kcal·mol^{-1})**	**log *A***
2.61	8.49

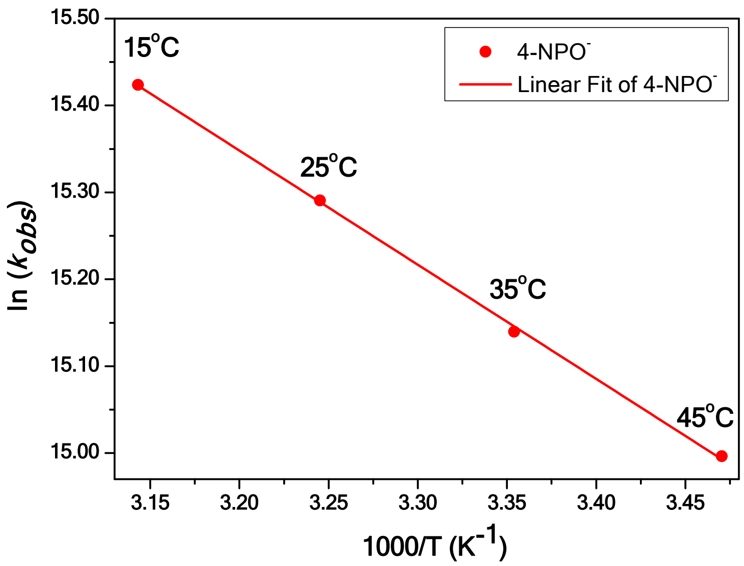

Figure S2. Arrhenius plot on proton transfer AcOH to 4-NPO- (red line).

Figure S3 shows linear fit for the activation parameters for 4-NPO- and shows the activation enthalpy (ΔH‡), entropy (ΔS‡) and Gibbs free energy (ΔG‡) at 25°C.

$$k_{obs} = \left(k_B T/h\right)e^{-(\Delta H^{\ddagger}/RT)}.e^{(\Delta S^{\ddagger}/R)}$$

$$\Delta G^{\ddagger} = \Delta H^{\ddagger} - T\Delta S^{\ddagger}$$

**ΔH‡ (kcal·mol-1)**	**ΔS‡ (cal·mol-1·K-1)**	**ΔG‡ (kcal·mol-1)**
2.01	-21.7	8.48

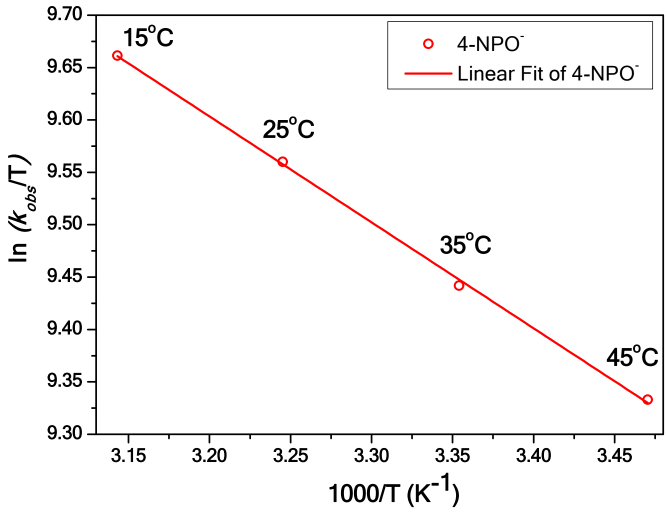

Figure S3. Effect of temperature on proton transfer AcOH to 4-NPO- (red line).
